# Temporal Trends in Phthalate Exposures: Findings from the National Health and Nutrition Examination Survey, 2001–2010

**DOI:** 10.1289/ehp.1306681

**Published:** 2014-01-15

**Authors:** Ami R. Zota, Antonia M. Calafat, Tracey J. Woodruff

**Affiliations:** 1 Department of Environmental and Occupational Health, School of Public Health and Health Services, George Washington University, Washington, DC, USA; 2 Program on Reproductive Health and the Environment, Department of Obstetrics, Gynecology, and Reproductive Sciences, University of California, San Francisco, Oakland, California, USA; 3 Division of Laboratory Sciences, National Center for Environmental Health, Centers for Disease Control and Prevention, Atlanta, Georgia, USA

## Abstract

Background: Phthalates are ubiquitous environmental contaminants. Because of potential adverse effects on human health, butylbenzyl phthalate [BBzP; metabolite, monobenzyl phthalate (MBzP)], di-*n*-butyl phthalate [DnBP; metabolite, mono-*n*-butyl phthalate (MnBP)], and di(2-ethylhexyl) phthalate (DEHP) are being replaced by substitutes including other phthalates; however, little is known about consequent trends in population-level exposures.

Objective: We examined temporal trends in urinary concentrations of phthalate metabolites in the general U.S. population and whether trends vary by sociodemographic characteristics.

Methods: We combined data on 11 phthalate metabolites for 11,071 participants from five cycles of the National Health and Nutrition Examination Survey (2001–2010). Percent changes and least square geometric means (LSGMs) were calculated from multivariate regression models.

Results: LSGM concentrations of monoethyl phthalate, MnBP, MBzP, and ΣDEHP metabolites decreased between 2001–2002 and 2009–2010 [percent change (95% CI): –42% (–49, –34); –17% (–23, –9); –32% (–39, –23) and –37% (–46, –26), respectively]. In contrast, LSGM concentrations of monoisobutyl phthalate, mono(3-carboxypropyl) phthalate (MCPP), monocarboxyoctyl phthalate, and monocarboxynonyl phthalate (MCNP) increased over the study period [percent change (95% CI): 206% (178, 236); 25% (8, 45); 149% (102, 207); and 15% (1, 30), respectively]. Trends varied by subpopulations for certain phthalates. For example, LSGM concentrations of ΣDEHP metabolites, MCPP, and MCNP were higher in children than adults, but the gap between groups narrowed over time (*p*_interaction_ < 0.01).

Conclusions: Exposure of the U.S. population to phthalates has changed in the last decade. Data gaps make it difficult to explain trends, but legislative activity and advocacy campaigns by nongovernmental organizations may play a role in changing trends.

Citation: Zota AZ, Calafat AM, Woodruff TJ. 2014. Temporal trends in phthalate exposures: findings from the National Health and Nutrition Examination Survey, 2001–2010. Environ Health Perspect 122:235–241; http://dx.doi.org/10.1289/ehp.1306681

## Introduction

Phthalate acid esters, also known as phthalates, are the predominant type of plasticizer used around the world. Low-molecular-weight phthalates, such as diethyl phthalate (DEP), di-*n*-butyl phthalate (DnBP), and diisobutyl phthalate (DiBP), are used in personal care products, solvents, adhesives, and medications [Kelley et al. 2012; Koniecki et al. 2011; U.S. Environmental Protection Agency (EPA) 2012]. High-molecular-weight phthalates, such as butylbenzyl phthalate (BBzP), di(2-ethylhexyl) phthalate (DEHP), diisononyl phthalate (DiNP), and diisodecyl phthalate (DiDP), are primarily used as plasticizers in polyvinyl chloride (PVC) applications found in building materials, cables and wires, toys, and food packaging (Schecter et al. 2013; Stringer et al. 2000; U.S. EPA 2012) (Table 1).

**Table 1:** Phthalates and urinary metabolites measured in the NHANES biomonitoring program.

Phthalate	Abbrev	Level of restriction in the U.S.^*a*^	MW^*b*^	Urinary metabolites	Abbrev	Common sources
Dimethyl phthalate	DMP	—	194.2	Monomethyl phthalate	MMP	Insect repellent, plastic bottles, food^*c,d*^
Diethyl phthalate	DEP	—	222.2	Monoethyl phthalate	MEP	Fragrance, cosmetics, medications^*e,f,g*^
Di-*n*-butyl phthalate	DnBP	++	278.4	Mono-*n*-butyl phthalate	MnBP	Cosmetics, medications, food packaging, food, PVC applications^*e,g,h,i,j,k*^
Diisobutyl phthalate	DiBP	—	278.3	Monoisobutyl phthalate	MiBP	Cosmetics, food, food packaging^*e,f,h,i*^
Butylbenzyl phthalate	BBzP	++	312.4	Monobenzyl phthalate Monobutyl phthalate (minor)	MBzP	PVC flooring, food, food packaging^*h,l*^
Dicyclohexyl phthalate	DCHP	—	330.4	Monocyclohexyl phthalate	MCHP	Food, food packaging^*h*^
Di(2-ethylhexyl) phthalate	DEHP	++	390.6	Mono(2-ethylhexyl) phthalate	MEHP	PVC applications, toys, cosmetics, food, food packaging^*e,f,h,i,j,k,m*^
				Mono(2-ethyl-5-hydroxyhexyl) phthalate	MEHHP	
				Mono(2-ethyl-5-oxohexyl) phthalate	MEOHP	
				Mono(2-ethyl-5-carboxypentyl) phthalate	MECPP	
Di-*n*-octyl phthalate	DnOP	+	390.6	Mono(3-carboxypropyl) phthalate	MCPP^*n*^	PVC applications, food, food packaging^*h,i,j*^
				Monooctyl phthalate	MOP	
Diisononyl phthalate	DiNP	+	418.6	Monoisononyl phthalate	MiNP	PVC applications, toys, flooring, wall covering^*j,m,o*^
Monocarboxyoctyl phthalate				MCOP		
Diisodecyl phthalate	DiDP	+	446.4	Monocarboxynonyl phthalate	MCNP	PVC applications, toys, wire and cables, flooring^*j,m,o*^
Abbreviations: —, no use restrictions; +, moderate use restrictions; ++, most use restrictions; abbrev, abbreviation. ^***a***^Indicates degree of risk management activities by federal and state governments in the United States (U.S. EPA 2012). ^***b***^We classified DMP, DEP, DnBP, and DiBP as low-molecular-weight phthalates, and BBzP, DCHP, DEHP, DnOP, DiNP, and DiDP high-molecular-weight phthalates. ^***c***^Karunamoorthi and Sabesan (2010). ^***d***^Al-Saleh et al. (2011). ^***e***^Koniecki et al. (2011). ^***f***^Dodson et al. (2012). ^***g***^Kelley et al. (2012). ^***h***^Fierens et al. (2012). ^***i***^Schecter et al. (2013). ^***j***^Kawakami et al. (2011). ^***k***^Cirillo et al. (2013). ^***l***^Kavlock et al. (2002). ^***m***^Stringer et al. (2000). MCPP is also a non­specific metabolite of several high-molecular-­weight phthalates. ^***o***^European Chemicals Agency (2012).						

Phthalates are not chemically bound to products and are therefore released into the environment where they may enter the human body via ingestion, inhalation, and dermal absorption (Meeker et al. 2009). Urinary metabolites of DEP, DnBP, BBzP, and DEHP have been widely detected in the U.S. population since 1999–2000, when phthalate metabolites were first systematically quantified in the National Health and Nutrition Examination Survey (NHANES) [Centers for Disease Control and Prevention (CDC) 2013; Silva et al. 2004; Woodruff et al. 2011]. Higher concentrations of some phthalate metabolites have been documented in certain sociodemographic subpopulations, including children (Koch et al. 2004; Wittassek et al. 2011), females (Silva et al. 2004; Trasande et al. 2013), nonwhite populations (Kobrosly et al. 2012; Trasande et al. 2013), and those of lower socioeconomic status (Kobrosly et al. 2012).

In animal studies, phthalates exhibit marked differences in toxicity depending on their chemical structure and timing of the exposure (Foster 2005; Gray et al. 2000; Howdeshell et al. 2008; National Research Council 2008; Parks et al. 2000). *In utero* exposure to certain phthalates, including BBzP, DnBP, and DEHP but not others (e.g., DEP), during the sexual differentiation period of rat development leads to reproductive tract malformations in androgen- and insulin-like 3 (*INSL3*)–dependent tissues (Barlow and Foster 2003; McKinnell et al. 2005; Wilson et al. 2004). Human epidemiologic studies have reported associations between exposure to DnBP, BBzP, and some other phthalates and adverse male reproductive outcomes, including reduced sperm quality, increased sperm DNA damage, and altered male genital development (Hauser et al. 2006, 2007; Meeker et al. 2009; Swan et al. 2005). Other studies have reported associations between gestational exposures to phthalates, including DEP, DnBP, BBzP, and DEHP, and outcomes suggesting impaired behavioral development (Braun et al. 2013; Engel et al. 2009; Swan et al. 2010; Whyatt et al. 2012).

Given the scientific community and public’s concern over phthalate toxicity, the European Union (EU) has banned the use of certain phthalates in toys, food-containing materials, and cosmetics (EU 2004, 2005, 2007). The U.S. federal government enacted legislation in 2008 that bans the use of DnBP, BBzP, and DEHP in any amount > 0.1% in child care articles including toys and placed an interim restriction on DiNP, DiDP, and di-*n*-octyl phthalate (DnOP) in toys that can be put in a child’s mouth [Consumer Product Safety Improvement Act (CPSIA) 2008; U.S. EPA 2012]. Although phthalate content in other products is not subject to legislative oversight in the United States, environmental and public health organizations have sought to reduce phthalate exposure by advocating for the removal of phthalates from personal care products and educating the public about how to find potentially safer alternatives (Campaign for Safe Cosmetics 2011).

Data on ingredient composition of consumer products are difficult to obtain because reporting is not required by law, but there is some evidence that the plasticizer market is changing. DEHP, which has historically been the most common phthalate plasticizer, is increasingly being substituted with DiNP and DiDP, and these two phthalates combined account for 30–60% of the current plasticizer market in the United States and the European Union (European Chemicals Agency 2012). Changes in reformulation and legislation may have important implications for phthalates exposures, and subsequent health risks, but until now, data were not available to assess temporal trends in phthalate exposures. Therefore, our study objective was to assess temporal trends in exposure to phthalates by analyzing changes in mean urinary concentrations of phthalate metabolites in the U.S. population between 2001 and 2010. In addition, we sought to assess whether temporal trends in urinary concentrations of phthalate metabolites differ by age, sex, race/ethnicity, or household income because these attributes have previously been correlated with phthalate exposures.

## Methods

*Study population*. We used data from the 2001–2002, 2003–2004, 2005–2006, 2007–2008, and 2009–2010 cycles of NHANES, a nationally representative survey and physical examination of the civilian, noninstitutionalized U.S. population conducted by the CDC. There were 13,288 participants with urinary measurements of phthalate metabolites and creatinine. We excluded participants who did not self-identify as non-Hispanic white, non-Hispanic black, or Mexican American (*n* = 1,460) and/or were missing information on household income (*n* = 902), resulting in a final sample size of 11,071 study participants.

*Phthalate metabolite measurements*. Phthalate metabolites are measured in approximately one-third of NHANES participants. Spot urine samples were collected in the Mobile Examination Center and stored at –20°C until shipped to the CDC’s National Center for Environmental Health (Atlanta, GA) for analysis. Concentrations of phthalate metabolites were quantified using solid phase extraction–high performance liquid chromatography–isotope dilution–tandem mass spectrometry (CDC 2013). Laboratory files were downloaded from the NHANES website in October 2012 and included the needed corrections for impurities in some of the previously used analytical standards (Langlois et al. 2012).

Fifteen phthalate metabolites have been measured in NHANES, but not all metabolites were measured in all cycles. The limit of detection (LOD) for a given metabolite often varied by cycle. To facilitate analysis across cycles, we assumed the maximal LOD for each metabolite in our analysis, and substituted values below the LOD with LOD divided by the square root of 2 because this method is used by the CDC (2013) and it produces reasonably nonbiased means and SDs (Hornung and Reed 1990). This report includes the 11 metabolites detected in more than 50% of the population in each cycle (LODs and detection frequencies are available in the Supplemental Material, Tables S1 and S2).

We calculated a summary metric for DEHP metabolites (ΣDEHP metabolites) equal to the molar sum of mono(2-ethylhexyl) phthalate (MEHP), mono(2-ethyl-5-hydroxyhexyl) phthalate (MEHHP), and mono(2-ethyl-5-oxohexyl) phthalate (MEOHP). [We omitted mono(2-ethyl-5-carboxypentyl) phthalate (MECPP) because it was not measured in 2001–2002.] We divided the concentrations of each metabolite by its molecular weight (MW) to obtain the molar equivalent (micromoles per liter) and then summed the concentrations in micromoles per liter to get total micromoles per liter of metabolites. To facilitate comparison with other analytes (Frederiksen et al. 2012; Wolff et al. 2010), we multiplied total micromoles per liter of metabolites by the average MW of the DEHP metabolites (MW = 288 μg/μmol) resulting in ΣDEHP metabolites concentrations expressed in nanograms per liter.

*Statistical analysis*. Analyses were conducted in SUDAAN, version 10.0 (Research Triangle Institute, Cary, NC). Because we combined five survey cycles, we calculated new sample weights for each participant according to the NHANES analytical guidelines (National Center for Health Statistics 2006) equal to one-fifth of the 2-year sample weights provided in the NHANES laboratory files. The degrees of freedom for our study sample equaled 77 and was calculated by subtracting the number of clusters in the first level of sampling (strata) from the number of clusters (PSUs, or primary sampling units) in the second level of sampling (National Center for Health Statistics 2006). Based on our degrees of freedom, we used a critical value of ±1.99 from the *t* distribution for the calculation of all confidence intervals. All analyses were adjusted for the nonrandom sampling design and the sample population weights.

We used multivariable regression models to assess the relationship between each phthalate metabolite concentration and time. For this analysis, we modeled NHANES sampling cycles using four indicator terms, with participants sampled in 2001–2002 as the reference group. Next, we constructed our “core” multivariable regression models where the outcome was phthalate metabolite concentrations and the independent variables were NHANES sampling cycle and urinary creatinine concentrations (to account for urinary dilution) (Barr et al. 2005). We natural log–transformed phthalate metabolite and creatinine data before regression analysis to account for their non-normal distributions. We examined residual diagnostics after transformation to assess these assumptions and tried various transformations of the data to assess the sensitivity of the conclusions to the assumptions of normality and equal variances.

From these regression models, we estimated *a*) percent changes in phthalate metabolite concentrations by NHANES cycle as [exp(β) – 1] × 100% with 95% CIs estimated as [exp(β ± 1.99 × SE) – 1] where β and SE are the estimated regression coefficient and standard error, respectively; and *b*) least squares geometric means (LSGMs) of phthalate metabolites concentrations by NHANES cycle as exp(least squares means) with 95% CIs as exp(least squares mean ± 1.99 × SE) where the least squares means is the cycle-specific mean of phthalate metabolite concentrations after adjusting for covariates. Next, we examined whether associations between NHANES sampling cycle and phthalate metabolites concentrations varied by age, sex, race/ethnicity, or household income. We first added the four demographic covariates to the “core” regression model described above. We then modeled multiplicative interactions between NHANES cycle and each demographic variable one at a time by adding product terms to the model for the interaction being evaluated, in addition to lower-order terms and covariates. LSGMs for subgroups presented in the main text were calculated from the multivariable models with the multiplicative interaction terms. Demographic variables were categorized as follows: age [children (6–11 years; *n* = 1,568), adolescents (12–19 years; *n* = 2,524), and adults (≥ 20 years; *n* = 6,979)]; sex [male (*n* = 5,524) and female (*n* = 5,547)]; race/ethnicity [non-Hispanic white (*n* = 5,305), non-Hispanic black (*n* = 2,951), and Mexican American (*n* = 2,815)]; and poverty–income ratio (PIR; the ratio of household income to poverty threshold adjusted to family size and inflation) [< 1 (i.e., beneath the poverty threshold; *n* = 2,604), 1–3 (*n* = 4,639), and > 3 (*n* = 3,828)]. This is a descriptive analysis, thus results for individual phthalates are not corrected for multiple comparisons. A (two-sided) *p*-value < 0.05 was considered statistically significant.

## Results

Concentrations of monobenzyl phthalate (MBzP), mono-*n*-butyl phthalate (MnBP), and monoethyl phthalate (MEP), metabolites of BBzP, DnBP, and DEP, respectively, were detected in at least 98% of participants in each cycle. The detection frequency of monoisobutyl phthalate (MiBP), a metabolite of DiBP, increased monotonically from 72% in 2001–2002 to 96% in 2009–2010. Concentrations of MEOHP, mono(3-carboxypropyl) phthalate (MCPP), monocarboxyoctyl phthalate (MCOP), monocarboxynonyl phthalate (MCNP), oxidative metabolites of DEHP, DnOP, DiNP, and DiDP, respectively, were detected in more than 89% of participants. (see Supplemental Material, Table S2; comparisons made using maximal LODs).

**Figure 1:**
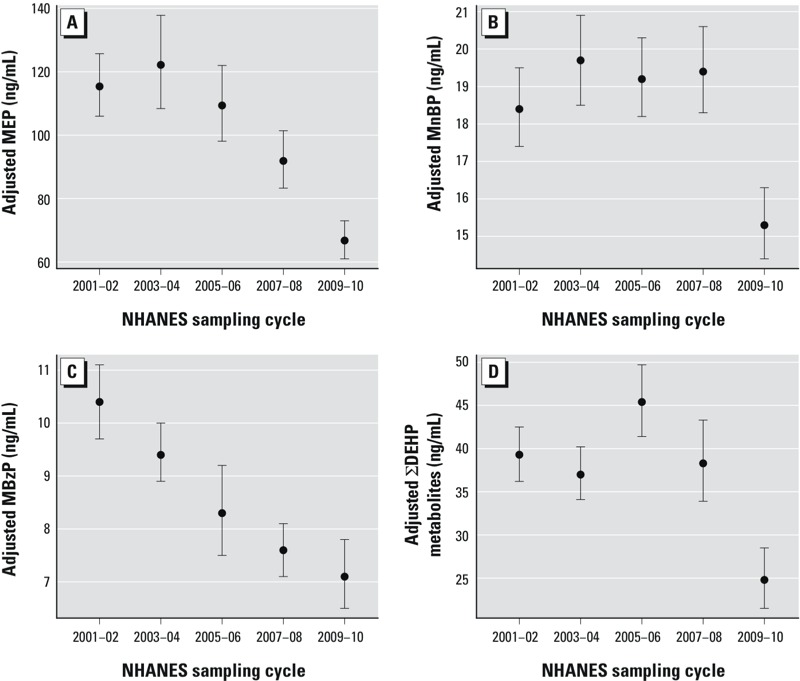
Association between phthalate metabolites and NHANES sampling cycle in the general U.S. population for (*A*) MEP (*n* = 11,071; parent phthalate = DEP) (*p* < 0.0001); (*B*) MnBP (*n* = 11,071; parent phthalate, DnBP; *p* < 0.0001); (*C*) MBzP (*n* = 11,071; parent phthalate, BBzP; *p* < 0.0001); and (*D*) ∑DEHP metabolites (*n* = 11,071; parent phthalate, DEHP; *p* < 0.0001). Models are adjusted for urinary creatinine. Data points represent LSGM and error bars represent 95% CIs. Corresponding numeric data are provided in Supplemental Material, Table S3. *p*-Value for the overall comparison between groups assessed by the Wald Test.

Concentrations of MEP, MnBP, MBzP, and the DEHP metabolites were significantly lower in 2009–2010 than in 2001–2002 (Figure 1; see also Supplemental Material, Table S3). LSGM concentrations of MEP declined monotonically between 2005 and 2010; compared with 2001–2002, LSGM concentrations were 20% (95% CI: –30, –9%) and 42% (95% CI: –49, –34%) lower in 2007–2008 and 2009–2010, respectively. LSGM concentrations of MBzP also steadily declined over time with the largest percent change [–32% (95% CI: –39, –23%)] observed between 2001–2002 and 2009–2010. There were no significant differences in LSGM concentrations of MnBP between 2001 and 2008, but 2009–2010 LSGMs were 17% lower (95% CI: –23, –9%) than those in 2001–2002. The temporal trend for ΣDEHP metabolites was nonmonotonic; LSGM concentrations of ΣDEHP metabolites increased from 39.3 ng/mL (95% CI: 36.3, 42.5) in 2001–2002 to 45.4 ng/mL (95% CI: 41.4, 49.7) in 2005–2006 and then decreased to 24.8 ng/mL (95% CI: 21.5, 28.5) in 2009–2010.

**Figure 2:**
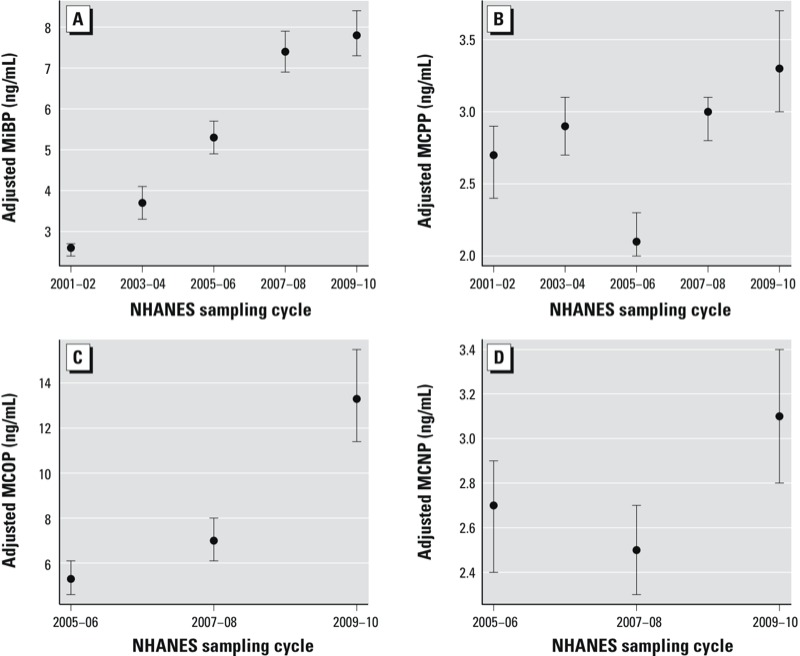
Association between phthalate metabolites and NHANES sampling cycle in the general U.S. population for (*A*) MiBP (*n* = 11,071; parent phthalate, DiBP; *p* < 0.0001); (*B*) MCPP (*n* = 11,071; parent phthalates, DnOP and a nonspecific metabolite of high-molecular-weight phthalates; *p* < 0.0001); (*C*) MCOP (*n* = 6,375; parent phthalate, DiNP; *p* < 0.0001); and (*D*) MCNP (*n* = 6,375; parent phthalate, DiDP; *p* = 0.004). Models are adjusted for urinary creatinine. Data points represent LSGM and error bars represent 95% CIs. Corresponding numeric data are provided in Supplemental Material, Table S3. *p*-Value for the overall comparison between groups assessed by the Wald Test.

Concentrations of MiBP, MCPP, MCOP, and MCNP were highest in 2009–2010 compared with earlier study cycles (Figure 2; see also Supplemental Material, Table S3). LSGM concentrations of MiBP monotonically increased over time and were 206% higher (95% CI: 178, 236%) in 2009–2010 compared with 2001–2002. LSGM concentrations of MCOP also monotonically increased over time and were 149% higher (95% CI: 102, 207%) in 2009–2010 compared with 2005–2006 (earliest cycle). For MCNP, LSGM concentrations were 15% higher (95% CI: 1, 30%) in 2009–2010 compared with 2005–2006 (earliest cycle) although LSGM concentrations in 2005–2006 and 2007–2008 were statistically similar. The trend in LSGM concentrations of MCPP was nonmonotonic with the lowest LSGM occurring in 2005–2006.

**Figure 3:**
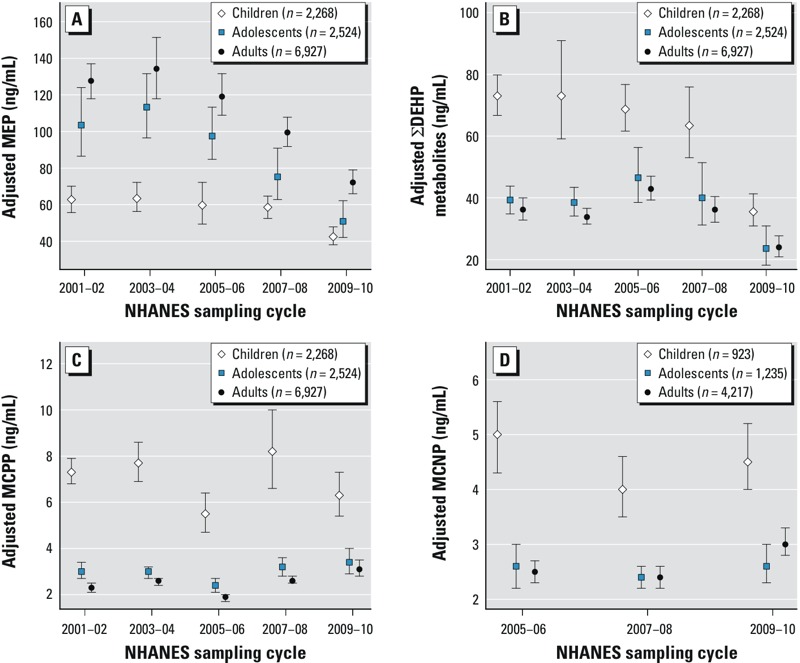
Association between phthalate metabolites and NHANES sampling cycle in the general U.S. population by age for (*A*) MEP (*p*_interaction_ = 0.04), (*B*) ∑DEHP metabolites (*p*_interaction_ = 0.002), (*C*) MCPP (*p*_interaction_ = 0.0004), and (*D*) MCNP (*p*_interaction_ = 0.009). Estimates are from linear regression models of interactions between NHANES sampling cycles and age adjusted for urinary creatinine, sex, race/ethnicity, and PIR. Data points represent LSGM and error bars represent 95% CIs. Corresponding numeric data are provided in Supplemental Material, Table S4.

Temporal trends varied by age for MEP, ΣDEHP metabolites, MCPP, and MCNP (Figure 3; see also Supplemental Material, Table S4). For MEP and ΣDEHP metabolites, all three age groups had significantly lower concentrations in 2009–2010 compared with 2001–2002 but children had some notable differences compared with that of adolescents and adults (*p*_interaction_ = 0.04 and 0.002 for MEP and ΣDEHP metabolites, respectively). Children had the lowest LSGM concentrations of MEP in all cycles with relatively stable exposures between 2001 and 2008; whereas LSGM concentrations of MEP in both adolescents and adults steadily declined after 2005–2006. For ΣDEHP metabolites, children had higher LSGM concentrations than adolescents and adults in all cycles but the differences between age groups narrowed over time. In addition, the temporal trend for ΣDEHP metabolites in adults and adolescents was nonmonotonic with highest LSGM concentrations in 2005–2006. For MCPP, the trend was non-monotonic for all three age groups, but among adults only, there was a statistically significant increase in LSGM concentrations between 2001–2002 and 2009–2010 (*p*_interaction_ = 0.0004). Similarly, LSGM concentrations of MCNP in 2009–2010 were higher than those in 2005–2006 (earliest cycle); whereas in children and adolescents, these metabolite concentrations were statistically similar over the study period (*p*_interaction_ = 0.009).

**Figure 4:**
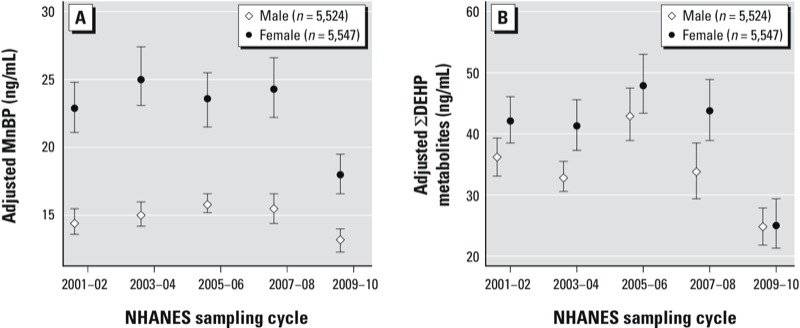
Association between phthalate metabolites and NHANES sampling cycle in the general U.S. population by sex for (*A*) MnBP (*p*_interaction_ = 0.03) and (*B*) ∑DEHP metabolites (*p*_interaction_ = 0.0001). Estimates are from linear regression models of interactions between NHANES sampling cycles and sex, adjusted for urinary creatinine, age (continuous), race/ethnicity, and PIR. Data points represent LSGM and error bars represent 95% CIs. Corresponding numeric data are provided in Supplemental Material, Table S5.

**Figure 5:**
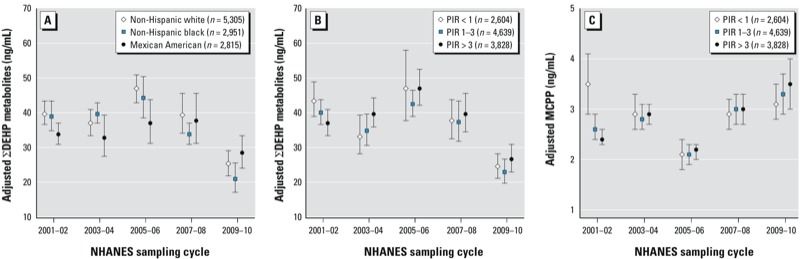
Association between phthalate metabolites and NHANES sampling cycle in the general U.S. population by race/ethnicity for ∑DEHP metabolites (*A*; *p*_interaction_ = 0.006) and by PIR for ∑DEHP metabolites (*B*; *p*_interaction_ = 0.01) and MCPP (*C*; *p*_interaction_ < 0.0001). Estimates in (*A*) are from linear regression models of interactions between NHANES sampling cycles and race/ethnicity adjusted for urinary creatinine, age (continuous), sex, and PIR. Estimates in (*B*) and (*C*) are from linear regression models of interactions between NHANES sampling cycles and PIR adjusted for urinary creatinine, age (continuous), sex, and race/ethnicity. Data points represent LSGM and error bars represent 95% CIs.

For MnBP and ΣDEHP metabolites, both sexes had significantly lower LSGMs in 2009–2010 compared with 2001–2002, but the percent decrease was greater in females than males (*p*_interaction_ = 0.03 and 0.0001, respectively) (Figure 4; see also Supplemental Material, Table S5). Trends also varied by race/ethnicity and PIR for ΣDEHP metabolites (*p*_interaction_ = 0.006 and 0.01, respectively) and by PIR for MCPP (*p*_interaction_ < 0.0001) (Figure 5). For example, the association between PIR and MCPP concentrations varied by cycle. In 2001–2002, participants with the lowest income (PIR < 1) had the highest LSGM concentrations of MCPP. However, in 2009–2010, income was inversely associated with MCPP concentrations and those with the highest income (PIR > 3) had a significantly higher LSGM of MCPP than those with the lowest income.

## Discussion

We observed pronounced changes in urinary concentrations of phthalate metabolites among the U.S. population between 2001 and 2010; urinary metabolite concentrations of DEP, DnBP, BBzP, and DEHP declined approximately 20–50%, whereas urinary metabolite concentrations of DiBP and DiNP increased by > 100%. To our knowledge, this is the first examination to date of temporal trends in phthalates exposures among a large, nationally representative sample of the U.S. population. Our findings are consistent with those from a German biomonitoring study that examined temporal trends over two decades in a convenience sample of predominately university students (age range, 20–29 years) (Wittassek et al. 2007).

Signiﬁcant data gaps make it difﬁcult to identify the underlying reasons for the observed trends in phthalate exposure with certainty. Although biomonitoring studies are useful for documenting population exposures to environmental chemicals, they are limited in their ability to identify the contribution of specific sources to personal exposure. Another data source that could provide insight on our findings is chemical production data available from the U.S. EPA (Chemical Data Reporting; http://epa.gov/cdr/). The data available for our study period suggest relatively stable trends in production for most phthalates (see Supplemental Material, Table S6). However, it is difficult to assess temporal trends in U.S. phthalate production with this data because chemical production by year is reported as a range (e.g., 100–500 million pounds/year) and not available on an annual basis but instead in 4- to 6-year intervals. In Germany, where more precise data is readily available, researchers report that a decline in production was accompanied by a decline in exposure to DnBP and DEHP (Wittassek et al. 2011).

As expected, we observed declines in metabolites of those phthalates that have been the focus of legislative activities, including bans on DnBP, BBzP, and DEHP in children’s products. However, legislative activity does not entirely explain the observed trends. For example, among the phthalates in the present study, we found the largest reductions in metabolite concentrations of DEP, a phthalate used in fragrances that is neither regulated in the United States or the European Union. In addition, metabolites of DnBP, BBzP, and DEHP were still detected in nearly all participants and the decline in DnBP metabolite concentrations was modest.

The success of advocacy efforts by public health and environmental organizations such as the Campaign for Safe Cosmetics (2014) may partly explain some of our findings. This campaign began in 2002 with a landmark report that documented widespread detection of DEP, DnBP, BBzP, and DEHP in the majority of beauty products tested (Houlihan et al. 2002). Over the last decade, it has used a multiprong strategy to reduce phthalate exposures from cosmetics by increasing consumer awareness of phthalate toxicity, creating a market for phthalate-free products, and pressuring the cosmetics industry to disclose chemical ingredients in their products (Campaign for Safe Cosmetics 2011). Although there are few data available on the extent of product reformulation in the United States, there is some evidence to suggest that the campaign’s activities have been influential in changing industry practices. For example, there has been an increased consumer demand for alternative products making it the fastest growing sector of the cosmetics market (Campaign for Safe Cosmetics 2011). Since 2004, more than 1,000 companies have pledged to remove chemicals of concern from personal care products and increase transparency of chemical ingredients in their products (Campaign for Safe Cosmetics 2011). In 2008, the coalition tested a subset of products originally examined in 2002 and found less frequent detection and lower concentrations of phthalates in most products (Campaign for Safe Cosmetics 2008). Our data suggest that reductions in DEP exposures have been the most pronounced, possibly because of changes in the formulation or use of personal care products, which are an important source of exposure to DEP (Duty et al. 2005; Just et al. 2010; Koch et al. 2013; Wormuth et al. 2006). Consistent with this hypothesis, metabolites of DnBP and DEHP declined less than DEP in NHANES over the study period. Diet is considered to be a principle route of DEHP exposure (Fromme et al. 2007; Koch et al. 2013; Wormuth et al. 2006), and DnBP exposures are not readily explained by either personal care product use or food-related sources (Duty et al. 2005; Fromme et al. 2007; Just et al. 2010; Koch et al. 2013). Future studies should examine how concentrations of individual phthalates in common exposure sources (such as building materials, cosmetics, and food) are changing over time. Moreover, future intervention efforts should consider aggregate sources of exposure if the goal is to reduce overall risk.

The rise in metabolite concentrations of DiBP and some high-molecular-weight phthalates suggest that manufacturers may be using them as substitutes for other phthalates even though the U.S. EPA has expressed concern about their use (U.S. EPA 2012), and there is an interim restriction on DnOP, DiNP, and DiDP in certain children’s toys (CPSIA 2008). DiBP is structurally similar to DnBP and may be a substitute for DnBP (Wittassek et al. 2007). DiNP and DiDP are replacing DEHP as a plasticizer in the global market (European Chemicals Agency 2012), including in the green or “safer alternatives” market (Dodson et al. 2012). For example, Dodson et al. (2012) measured chemical ingredients in conventional and alternative consumer products purchased in 2007 and detected DiNP in alternative products only. Similarly, they detected DiBP but not DnBP in nail polish samples. Toxicological studies suggest that DiBP and DiNP may disrupt androgen signaling and act cumulatively with other phthalates to affect male reproductive end points (National Research Council 2008). Although epidemiologic evidence of these replacement phthalates is limited, a recent cross-sectional study of 623 Norwegian children (Bertelsen et al. 2013) reported associations between current asthma and urinary metabolites of DiNP and DiDP, but not with any of the other phthalate metabolites. Given the likely increase in human exposure to replacement phthalates, further study on their adverse health effects in epidemiologic studies is warranted.

Our findings also suggest that temporal trends in phthalates exposure are not uniform across the population and that subpopulations with the highest initial phthalates exposures often experienced the greatest decline over the study period. For example, we observed a more rapid decline in DEP metabolite concentrations in adults and adolescents compared with children, possibly reflecting differences in personal care product use. We also found a greater decline in concentrations of DnBP and DEHP metabolites among females than males, potentially reflecting differences in exposure sources or behavior. For example, a Swiss study of 1,215 participants found that women have a higher risk perception of chemicals and a stronger preference for natural food than men (Dickson-Spillmann et al. 2011). For high-molecular-weight phthalates such as DEHP and DiDP, metabolite concentrations were higher in children than adults, but the gap between groups narrowed over time. These phthalates are commonly used in PVC applications including toys, and the larger reductions among children may reflect the legislative emphasis on limiting phthalates in children’s toys.

There are several key strengths to our study. NHANES provides an unparalleled opportunity to document changes in environmental chemicals exposures because each survey captures a large, nationally representative sample of the general U.S. population that is diverse with respect to geography, age, race/ethnicity, and income. The large and diverse sample allows for statistically reliable assessment of trends in demographic subgroups.

Our main study limitation is the cross-sectional design of NHANES that inhibits examination of longitudinal changes in phthalates metabolite concentrations in the same participants. Also, NHANES does not measure phthalate metabolites in children < 6 years of age. Our findings may not be generalizable to young children, who may experience different exposures to some phthalates than older children as a result of their higher food consumption related to body weight, higher dust ingestion from their playing habits, and distinct mouthing behavior (Becker et al. 2009; Wittassek et al. 2011); children also possess different behaviors and physiology than adults (U.S. EPA 2008). Additional biomonitoring studies in young children may be warranted because of their potentially higher susceptibility to the adverse effects of environmental stressors and because young children are likely to be most impacted by regulations limiting phthalate content in toys. There may be false positives due to the large number of models evaluated. However, all of our main findings (presented in Figures 1 and 2) and half of the interaction models would pass Bonferroni correction for multiple testing. Last, although the CDC conducted all phthalate metabolite measurements, modifications were made to the analytical methods between cycles that may affect the frequency of detection of the measured metabolites. To account for some of these differences, we applied the same LOD to each cycle.

## Conclusions

Our analysis of biomonitoring data from a nationally representative sample suggests that U.S. population exposure to phthalates has changed in the last decade. Although exposures to DnBP, BBzP, and DEHP have declined, exposures to replacement phthalates such as DiNP and DiBP have increased. The observed temporal trends are difficult to explain because of significant data gaps, but may at least partly reflect the effects of legislative activity and the advocacy efforts of nongovernmental organizations on consumer behavior and the use of phthalates in consumer products.

## Supplemental Material

(283 KB) PDF
