# Supplementary material for: Temporal Trends in Phthalate Exposures: Findings from the National Health and Nutrition Examination Survey, 2001–2010
Source: Environ Health Perspect. 2014 Jan 15;122(3):235–41. doi: 10.1289/ehp.1306681 (PMC3948032; doi:10.1289/ehp.1306681)
Supplement: (283 KB) PDF [file ehp.1306681.s001.pdf]

Environmental Health Perspectives doi:10.1289/ehp.1306681

Supplemental Material

Temporal Trends in Phthalate Exposures: Findings from the National Health and Nutrition Examination Survey, 2001–2010

Ami R. Zota, Antonia M. Calafat, and Tracey J. Woodruff

Table of Contents

Supplemental Material, Table S1……………………………………..……….Page 2

Supplemental Material, Table S2……………………………………….……..Page 3

Supplemental Material, Table S3……………………………………….….Page 4

Supplemental Material, Table S4……………………………………….……..Page 6

Supplemental Material, Table S5……………………………………….…..Page 8

Supplemental Material, Table S6……………………………………………...Page 9

Supplemental Material, Table S1. Limits of detection (LOD) for urinary phthalate metabolites by NHANES cycle.

Parent compound: *Phthalate metabolite*	2001-02 (ng/mL)	2003-04 (ng/mL)	2005-06 (ng/mL)	2007-08 (ng/mL)	2009-10 (ng/mL)	Maximum LOD (ng/mL)
**Dimethyl phthalate (DMP)**						
Monomethyl phthalate (MMP)	0.2	1.0	1.1	1.1	0.5	1.1
**Diethyl phthalate (DEP)**						
Monoethyl phthalate (MEP)[a]	0.6	0.3	0.5	0.5	0.5	0.6
**Di-*n*-butyl phthalate (DnBP)**						
*Mono-*n*-butyl phthalate (MnBP)*[a]	1.1	0.4	0.6	0.6	0.4	1.1
**Di-iso-butyl phthalate (DiBP)**						
Monoisobutyl phthalate (MiBP)[a]	1.0	0.3	0.3	0.3	0.2	1.0
**Butylbenzyl phthalate (BBzP)**						
Monobenzyl phthalate (MBzP)[a]	0.2	0.1	0.2	0.2	0.2	0.2
**Dicyclohexyl phthalate (DCHP)**						
Monocyclohexyl phthalate (MCHP)	0.6	0.4	0.6	0.6	0.4	0.6
**Di (2-ethylhexyl) phthalate (DEHP)**						
Mono(2-ethylhexyl) phthalate (MEHP)[a]	1.0	0.9	1.2	1.1	0.5	1.2
Mono(2-ethyl-5-hydroxyhexyl) phthalate (MEHHP)[a]	1.0	0.3	0.7	0.7	0.2	1.0
Mono(2-ethyl-5-oxohexyl) phthalate (MEOHP) [a]	1.1	0.5	0.7	0.6	0.2	1.1
Mono(2-ethyl-5-carboxypentyl) phthalate (MECPP)[a]	--	0.3	0.6	0.5	0.2	0.6
**Di-*n*-octyl phthalate (DnOP)**						
Mono(3-carboxypropyl) phthalate (MCPP)[a]	0.4	0.2	0.2	0.2	0.2	0.4
Monooctyl phthalate (MOP)	1.7	1.7	1.9	1.9	0.8	1.9
**Di-iso-nonyl phthalate (DiNP)**						
Monoisononyl phthalate (MiNP)	1.2	1.5	1.2	1.2	0.8	1.5
Monocarboxyoctyl phthalate (MCOP)[a]	--	--	0.7	0.7	0.2	0.7
**Di-iso-decyl phthalates (DiDP)**						
Monocarboxynonyl phthalate (MCNP)[a]	--	--	0.6	0.5	0.2	0.6

[a]Indicates phthalate metabolites included in the present study.

Supplemental Material, Table S2. Detection frequency (%) of phthalate metabolites by NHANES sampling cycle using maximal LODs (N = 11071).

**Parent compound:** ***Phthalate metabolite***	**2001-02 (n = 2418)**	**2003-04 (n = 2278)**	**2005-06 (n = 2243)**	**2007-08 (n = 2040)**	**2009-10 (n = 2092)**	**P-value[a]**
**Dimethyl phthalate (DMP)**						
Monomethyl phthalate (MMP)	60	54	37	38	45	< 0.0001
**Diethyl phthalate (DEP)**						
Monoethyl phthalate (MEP)[b]	100	100	100	100	100	0.17
**Di-*n*-butyl phthalate (DnBP)**						
*Mono-*n*-butyl phthalate (MnBP)[b]*	98	98	99	98	98	0.73
**Di-iso-butyl phthalate (DiBP)**						
Monoisobutyl phthalate (MiBP)[b]	72	88	89	94	96	< 0.0001
**Butylbenzyl phthalate (BBzP)**						
Monobenzyl phthalate (MBzP)[b]	99	99	98	98	99	0.06
**Dicyclohexyl phthalate (DCHP)**						
Monocyclohexyl phthalate (MCHP)	15	6	2	4	2	< 0.0001
**Di (2-ethylhexyl) phthalate (DEHP)**						
Mono(2-ethylhexyl) phthalate (MEHP)[b]	78	61	67	66	56	< 0.0001
Mono(2-ethyl-5-hydroxyhexyl) phthalate (MEHHP)[b]	98	98	99	99	98	0.04
Mono(2-ethyl-5-oxohexyl) phthalate (MEOHP)[b]	96	97	98	96	96	0.01
Mono(2-ethyl-5-carboxypentyl) phthalate (MECPP)	--	100	100	100	100	0.36
**Di-*n*-octyl phthalate (DnOP)**						
Mono(3-carboxypropyl) phthalate (MCPP)[b]	93	96	93	95	95	0.0008
Monooctyl phthalate (MOP)	2	1	1	1	1	0.05
**Di-iso-nonyl phthalate (DiNP)**						
Monoisononyl phthalate (MiNP)	4	5	11	10	28	< 0.0001
Monocarboxyoctyl phthalate (MCOP)[b]	--	--	96	96	98	0.007
**Di-iso-decyl phthalates (DiDP)**						
Monocarboxynonyl phthalate (MCNP)[b]	--	--	89	90	94	0.004

[a]P-value for overall comparison of groups evaluated by chi-square test of independence. [b]Indicates phthalate metabolites included in the present study.

Supplemental Material, Table S3. Association between phthalate metabolites concentrations (ng/ml) and NHANES cycle in the US general population after adjustment for urinary creatinine concentrations, calculated from multivariable regression models (N = 11071).[a]

Metabolite		2001-2002 (n = 2418)	2003-2004 (n = 2278)	2005-2006 (n = 2243)	2007-2008 (n = 2040)	2009-2010 (n = 2092)	P-value[b]
MEP	% change (95% CI)	*(ref)*	6 (-9, 23)	-5 (-17, 9)	-20 (-30, -9)	-42 (-49, -34)	< 0.0001
	LSGM (95% CI)	115.4 (106.0, 125.7)	122.2 (108.4, 137.8)	109.4 (98.1, 122)	91.9 (83.3, 101.4)	66.8 (61.0, 73.0)	
MnBP	% change (95% CI)	*(ref)*	7 (-1, 16)	5 (-3,13)	5 (-3,14)	-17 (-23, -9)	< 0.0001
	LSGM (95% CI)	18.4 (17.4, 19.5)	19.7 (18.5, 20.9)	19.2 (18.2, 20.3)	19.4 (18.3, 20.6)	15.3 (14.4, 16.3)	
MiBP	% change (95% CI)	*(ref)*	43 (27, 62)	106 (87, 127)	188 (163, 216)	206 (178, 236)	< 0.0001
	LSGM (95% CI)	2.6 (2.4, 2.7)	3.7 (3.3, 4.1)	5.3 (4.9, 5.7)	7.4 (6.9, 7.9)	7.8 (7.3, 8.4)	
MBzP	% change (95% CI)	*(ref)*	-9 (-17, -1)	-20 (-29, -9)	-27 (-34, -20)	-32 (-39, -23)	< 0.0001
	LSGM (95% CI)	10.4 (9.7, 11.1)	9.4 (8.9, 10.0)	8.3 (7.5, 9.2)	7.6 (7.1, 8.1)	7.1 (6.5, 7.8)	
MEHP	% change (95% CI)	*(ref)*	-42 (-49, -34)	-29 (-37,-20)	-36 (-45, -25)	-53 (-59, -45)	< 0.0001
	LSGM (95% CI)	4.1 (3.7, 4.6)	2.4 (2.2, 2.6)	3.0 (2.8, 3.2)	2.6 (2.4, 3.0)	2.0 (1.7, 2.2)	
MEHHP	% change (95% CI)	*(ref)*	5 (-7, 18)	31 (15, 50)	16 (-1, 35)	-29 (-40, -15)	< 0.0001
	LSGM (95% CI)	19.5 (17.8, 21.3)	20.4 (18.8, 22.1)	25.6 (23.2, 28.2)	22.5 (19.9, 25.6)	13.8 (11.9, 16.1)	
MEOHP	% change (95% CI)	*(ref)*	4 (-8, 17)	25 (10, 42)	-4 (-17, 12)	-34 (-43, -22)	< 0.0001
	LSGM (95% CI)	13.1 (12.0, 14.2)	13.6 (12.5, 14.9)	16.4 (14.9, 18.0)	12.6 (11.1, 14.2)	8.7 (7.6, 10.0)	
MECPP[c]	% change (95% CI)	--	*(ref)*	-14 (-1, 31)	-2 (-17, 17)	-40 (-48, -30)	< 0.0001
	LSGM (95% CI)	--	33.9 (30.6, 37.5)	38.6 (34.9, 42.6)	33.2 (28.9, 38.2)	20.4 (18.1, 23.0)	
∑DEHP metabolites[d]	% change (95% CI)	*(ref)*	-6 (-16, 6)	16 (2, 30)	-3 (-16, 13)	-37 (-46, -26)	< 0.0001
	LSGM (95% CI)	39.3 (36.3, 42.5)	37.0 (34.1, 40.2)	45.4 (41.4, 49.7)	38.3 (33.9, 43.3)	24.8 (21.5, 28.5)	
MCPP	% change (95% CI)	*(ref)*	9 (-2, 21)	-20 (-29, -10)	11 (0, 24)	25 (8, 45)	< 0.0001
	LSGM (95% CI)	2.7 (2.4, 2.9)	2.9 (2.7, 3.1)	2.1 (2.0, 2.3)	3.0 (2.8, 3.1)	3.3 (3.0, 3.7)	
MCOP[e]	% change (95% CI)	--	--	*(ref)*	31 (8, 58)	149 (102, 207)	< 0.0001
	LSGM (95% CI)	--	--	5.3 (4.6, 6.1)	7.0 (6.1, 8.0)	13.3 (11.4, 15.5)	
MCNP[e]	% change (95% CI)	--	--	*(ref)*	-7 (-18, 5)	15 (1, 30)	0.004
	LSGM (95% CI)	--	--	2.7 (2.4, 2.9)	2.5 (2.3, 2.7)	3.1 (2.8, 3.3)	

[a]The percent changes and least square geometric means (LSGMs) were calculated from multivariate regression models where the dependent variables were natural log-transformed phthalate metabolite concentrations and the independent variables were NHANES cycle (using four indicator terms and 2001-2002 participants as the reference group) and natural log-transformed urinary creatinine concentrations. We estimated the percent change in phthalate metabolite concentrations by NHANES cycle as $(e^{(\beta)}-1) * 100\%$ with 95% confidence intervals (CIs) estimated

as $(e^{(\beta \pm 1.99 * SE)} - 1)$ where β and SE are the estimated regression coefficient and standard error, respectively. We estimated least squares geometric means (LSGMs) of phthalate metabolites concentrations by NHANES cycle as $e^{(\text{least squares means})}$ with 95% CIs as $e^{(\text{least squares mean} \pm 1.99 * SE)}$ where the least squares means are the cycle-specific means of phthalate metabolite concentrations after adjustment for covariates. We used a critical value of ±1.99 from the t distribution for the calculation of all confidence intervals. [b]P-value for the overall comparison between groups assessed by the Wald Test. [c]Not measured in NHANES 2001-2002. [d]∑DEHP metabolites (expressed as ng/mL) is the molar sum of MEHP (MW = 278), MEHHP (MW = 294), and MEOHP (MW = 292) multiplied by the average MW of the three metabolites (MW = 288). [e]Not measured in NHANES 2001-2002 or 2003-2004.

Supplemental Material, Table S4. Association (LSGM (95% CI) between phthalate metabolites and NHANES sampling cycle in the US general population by age in the US general population (N = 11071)[a].

		2001-2002 (n = 2418)	2003-2004 (n = 2278)	2005-2006 (n = 2243)	2007-2008 (n = 2040)	2009-2010 (n = 2092)	$P_{interaction}$ [b]
MEP							0.04
Children	% change (95% CI)	*(ref)*	2 (-14, 21)	-4 (-24, 20)	-7 (-21, 9)	-32 (-42, -20)	
	LSGM (95% CI)	62.8 (55.7, 70.1)	63.4 (56.3, 72.2)	59.7 (49.4, 72.2)	58.6 (52.5, 64.7)	42.5 (42.1, 62.2)	
Adolescents	% change (95% CI)	*(ref)*	8 (-14, 38)	-6 (-25, 19)	-27 (-43, -6)	-51 (-62, -36)	
	LSGM (95% CI)	103.5 (86.5, 124.0)	113.3 (96.5, 131.6)	97.5 (84.8, 113.3)	75.2 (62.8, 90.9)	50.9 (42.1, 62.2)	
Adults	% change (95% CI)	*(ref)*	5 (-10, 22)	-6 (-17, 6)	-22 (-30, -13)	-43 (-49, -36)	
	LSGM (95% CI)	127.7 (117.9, 137.0)	134.3 (117.9, 151.4)	119.1 (108.9, 131.6)	99.5 (91.8, 107.8)	72.2 (66.0, 79.0)	
∑DEHP metabolites[c]							0.002
Children	% change (95% CI)	*(ref)*	0 (-21, 26)	-7 (-19, 8)	-13 (-29, 6)	-51 (-59, -42)	
	LSGM (95% CI)	73.0 (66.7, 79.8)	73.0 (59.1, 90.9)	68.7 (61.6, 76.7)	63.4 (53.0,75.9)	35.5 (30.9, 41.3)	
Adolescents	% change (95% CI)	*(ref)*	-1 (-16, 16)	19 (-4, 48)	2 (-23, 35)	-39 (-55, -20)	
	LSGM (95% CI)	39.3 (34.8, 43.8)	38.5 (34.1, 43.4)	46.5 (38.5, 56.3)	40.0 (31.2, 51.4)	23.6 (18.2, 30.9)	
Adults	% change (95% CI)	*(ref)*	-7 (-17, 6)	19 (4, 36)	-1 (-15, 16)	-34 (-45, -21)	
	LSGM (95% CI)	36.2 (32.8, 40.0)	33.8 (31.5, 36.6)	42.9 (39.3, 47.0)	36.2 (32.1, 40.4)	24.0 (20.9, 27.7)	
MCPP							0.0004
Children	% change (95% CI)	*(ref)*	5 (-9, 21)	-25 (-36, -11)	11 (-10, 38)	-14 (-27, 2)	
	LSGM (95% CI)	7.3 (6.8, 7.9)	7.7 (6.9, 8.6)	5.5 (4.7, 6.4)	8.2 (6.6, 10.0)	6.3 (5.4, 7.3)	
Adolescents	% change (95% CI)	*(ref)*	-2 (-14, 11)	-22 (-33, -9)	4 (-12, 22)	12 (-9, 35)	
	LSGM (95% CI)	3.0 (2.7, 3.4)	3.0 (2.7, 3.2)	2.4 (2.1, 2.7)	3.2 (2.8, 3.6)	3.4 (2.9, 4.0)	
Adults	% change (95% CI)	*(ref)*	12 (0, 26)	-18 (-27, -8)	15 (3, 28)	36 (19, 57)	
	LSGM (95% CI)	2.3 (2.1, 2.5)	2.6 (2.4, 2.7)	1.9 (1.7, 2.0)	2.6 (2.5, 2.8)	3.1 (2.8, 3.5)	
MCNP[d]							0.009
Children	% change (95% CI)	--	--	*(ref)*	-19 (-33, -2)	-8 (-23, 9)	
	LSGM (95% CI)	--	--	5.0 (4.3, 5.6)	4.0 (3.5, 4.6)	4.5 (4.0, 5.2)	
Adolescents	% change (95% CI)	--	--	*(ref)*	-7 (-21, 9)	2 (-16, 25)	
	LSGM (95% CI)	--	--	2.6 (2.2, 3.0)	2.4 (2.2, 2.6)	2.6 (2.3, 3.0)	
Adults	% change (95% CI)	--	--	*(ref)*	-4 (-16, 9)	21 (6, 38)	
	LSGM (95% CI)	--	--	2.5 (2.3, 2.7)	2.4 (2.2, 2.6)	3.0 (2.8, 3.3)	

[a]Estimates are from linear regression models of interactions between NHANES sampling cycles and age adjusted for urinary creatinine, sex, race/ethnicity, and PIR. The least square geometric means (LSGMs) were calculated from multivariate regression models where the dependent variables were natural log-

transformed phthalate metabolite concentrations and the independent variables were NHANES cycle (using four indicator terms and 2001-2002 participants as the reference group) and natural log-transformed urinary creatinine concentrations. We estimated the percent change in phthalate metabolite concentrations by NHANES cycle as $(e^{(\beta)}-1) * 100\%$ with 95% confidence intervals (CIs) estimated as $(e^{(\beta \pm 1.99 * SE)}-1)$ where β and SE are the estimated regression coefficient and standard error, respectively. We estimated least squares geometric means (LSGMs) of phthalate metabolites concentrations by NHANES cycle as $e^{(\text{least squares means})}$ with 95% CIs as $e^{(\text{least squares mean} \pm 1.99 * SE)}$ where the least squares means are the cycle-specific means of phthalate metabolite concentrations after adjustment for covariates. We used a critical value of ±1.99 from the t distribution for the calculation of all confidence intervals. [b]P-value for the overall comparison between groups assessed by the Wald Test. [c]∑DEHP metabolites (expressed as ng/mL) is the molar sum of MEHP (MW = 278), MEHHP (MW = 294), and MEOHP (MW = 292) multiplied by the average MW of the three metabolites (MW = 288). [d]Not measured in NHANES 2001-2002 or 2003-2004.

Supplemental Material, Table S5. Association (LSGM (95% CI) between phthalate metabolites and NHANES sampling cycle in the US general population by sex in the US general population (N = 11071).[a]

		**2001-2002 (n = 2418)**	**2003-2004 (n = 2278)**	**2005-2006 (n = 2243)**	**2007-2008 (n = 2040)**	**2009-2010 (n = 2092)**	$\mathbf{P_{interaction}}$[b]
MnBP							0.03
Males	% change (95% CI)	*(ref)*	4 (-6, 15)	9 (1, 20)	7 (-3, 19)	-10 (-17, 0)	
	LSGM (95% CI)	14.6 (13.6, 15.6)	15.2 (14.2, 16.1)	16.0 (15.3, 16.8)	15.6 (14.6, 16.8)	13.2 (12.4, 14.2)	
Females	% change (95% CI)	*(ref)*	11 (-2, 23)	3 (-9, 16)	6 (-7, 20)	-21 (-30, -12)	
	LSGM (95% CI)	22.6 (20.9, 24.5)	25.0 (22.9, 27.1)	23.3 (21.3, 25.5)	24.0 (21.8, 26.3)	17.8 (16.4, 19.3)	
∑DEHP metabolites[c]							0.0001
Males	% change (95% CI)	*(ref)*	8 (-18, 4)	20 (4, 36)	-6 (21, 12)	-31 (-41, -19)	
	LSGM (95% CI)	36.2 (32.8, 39.6)	33.1 (30.9, 35.9)	42.9 (38.9, 47.5)	33.8 (29.7, 38.9)	25.0 (22.0, 28.5)	
Females	% change (95% CI)	*(ref)*	-2 (-15, 14)	14 (0, 31)	4 (-10, 21)	-41 (-51, -27)	
	LSGM (95% CI)	41.7 (38.1, 46.1)	41.3 (36.6, 46.1)	47.9 (43.4, 52.5)	43.4 (38.5, 48.9)	25.0 (21.1, 29.4)	

[a]Estimates are from linear regression models of interactions between NHANES sampling cycles and sex adjusted for urinary creatinine, age (continuous), race/ethnicity, and PIR. The least square geometric means (LSGMs) were calculated from multivariate regression models where the dependent variables were natural log-transformed phthalate metabolite concentrations and the independent variables were NHANES cycle (using four indicator terms and 2001-2002 participants as the reference group) and natural log-transformed urinary creatinine concentrations. We estimated the percent change in phthalate metabolite concentrations by NHANES cycle as $(e^{(\beta)}-1) * 100\%$ with 95% confidence intervals (CIs) estimated as $(e^{(\beta \pm 1.99 * SE)}-1)$ where β and SE are the estimated regression coefficient and standard error, respectively. We estimated least squares geometric means (LSGMs) of phthalate metabolites concentrations by NHANES cycle as $e^{(\text{least squares means})}$ with 95% CIs as $e^{(\text{least squares mean} \pm 1.99 * SE)}$ where the least squares means are the cycle-specific means of phthalate metabolite concentrations after adjustment for covariates. We used a critical value of ±1.99 from the t distribution for the calculation of all confidence intervals. [b]P-value for the overall comparison between groups assessed by the Wald Test. [c]∑DEHP metabolites (expressed as ng/mL) is the molar sum of MEHP (MW =278), MEHHP (MW = 294), and MEOHP (MW = 292) multiplied by the average MW of the three metabolites (MW = 288).

Supplemental Material, Table S6. US chemical production data (pounds per year) by reporting year as provided by the US EPA.[a]

Species	Cas No.	1990	1994	1998	2002	2006	2012
BBzP	85-68-7	100 – 500 M	50 – 100 M	100 – 500 M	50 – 100 M	50 – 100 M	50 – 100 M
DEHP	117-81-7	100 – 500 M	100 – 500 M	100 – 500 M	100 – 500 M	100 – 500 M	153 M
DnBP	84-74-2	10 – 50 M	10 – 50 M	10 – 50 M	10 – 50 M	10 – 50 M	7 M
DiBP	84-69-5	1 – 10 M	1 – 10 M	1 – 10 M	0.5 – 1 M	0.5 – 1 M	0.5 M
DEP	84-66-2	10 – 50 M	10 – 50 M	10 – 50 M	10 – 50 M	10 – 50 M	6 M
DNOP	117-84-0	1 – 10 M	1 – 10 M	1 – 10 M	1 – 10 M	10 – 50 M	Confidential
DINP 1	68515-48-0	> 1B	100 – 500 M	100 – 500 M	100 – 500 M	100 – 500 M	100 – 250 M
DINP 2	28553-12-0	10 – 50 M	10 – 50 M	10 – 50 M	10 – 50 M	100 – 500 M	N/A
DIDP 1	26761-40-0	50 – 100 M	50 – 100 M	10 – 50 M	1 – 10 M	1 – 10 M	50 – 100 M
DIDP 2	68515-49-1	100 – 500 M	100 – 500 M	100 – 500 M	100 – 500 M	100 – 500 M	163 M

[a]Data obtained through the non-confidential US EPA Chemical Data Reporting Program. M = million, B = billion.

More information at http://epa.gov/cdr/
